# Typhoidal *Salmonella* human challenge studies: ethical and practical challenges and considerations for low-resource settings

**DOI:** 10.1186/s13063-019-3844-z

**Published:** 2019-12-19

**Authors:** Meriel Raymond, Malick M. Gibani, Nicholas P. J. Day, Phaik Yeong Cheah

**Affiliations:** 1 Oxford Vaccine Group Centre for Clinical Vaccinology and Tropical Medicine (CCVTM), Churchill Hospital, Old Road, Headington, Oxford, OX3 7LE UK; 2 Mahidol Oxford Tropical Medicine Research Unit (MORU), Faculty of Tropical Medicine, Mahidol University, 420/6 Rajvithi Road, Bangkok, 10400 Thailand; 3 Nuffield Department of Clinical Medicine, Centre for Tropical Medicine and Global Health, University of Oxford, Old Road Campus, Roosevelt Drive, Oxford, OX3 7FZ UK; 4 Nuffield Departmemt of Population Health, The Ethox Centere, University of Oxford, Old Road, Oxford, OX3 7LF UK

**Keywords:** Research ethics, Controlled human infection models, Low-resource settings, Typhoidal *Salmonella*

## Abstract

Typhoidal *Salmonella* is a major global problem affecting more than 12 million people annually. Controlled human infection models (CHIMs) in high-resource settings have had an important role in accelerating the development of conjugate vaccines against *Salmonella* Typhi.

The typhoidal *Salmonella* model has an established safety profile in over 2000 volunteers in high-income settings, and trial protocols, with modification, could be readily transferred to new study sites. To date, a typhoidal *Salmonella* CHIM has not been conducted in a low-resource setting, although it is being considered.

Our article describes the challenges posed by a typhoidal *Salmonella* CHIM in the high-resource setting of Oxford and explores considerations for an endemic setting.

Development of CHIMs in endemic settings is scientifically justifiable as it remains unclear whether findings from challenge studies performed in high-resource non-endemic settings can be extrapolated to endemic settings, where the burden of invasive *Salmonella* is highest. Volunteers are likely to differ across a range of important variables such as previous *Salmonella* exposure, diet, intestinal microbiota, and genetic profile. CHIMs in endemic settings arguably are ethically justifiable as affected communities are more likely to gain benefit from the study. Local training and research capacity may be bolstered.

Safety was of primary importance in the Oxford model. Risk of harm to the individual was mitigated by careful inclusion and exclusion criteria; close monitoring with online diary and daily visits; 24/7 on-call staffing; and access to appropriate hospital facilities with capacity for in-patient admission. Risk of harm to the community was mitigated by exclusion of participants with contact with vulnerable persons; stringent hygiene and sanitation precautions; and demonstration of clearance of *Salmonella* infection from stool following antibiotic treatment.

Safety measures should be more stringent in settings where health systems, transport networks, and sanitation are less robust.

We compare the following issues between high- and low-resource settings: scientific justification, risk of harm to the individual and community, benefits to the individual and community, participant understanding, compensation, and regulatory requirements.

We conclude that, with careful consideration of country-specific ethical and practical issues, a typhoidal *Salmonella* CHIM in an endemic setting is possible.

## Background

Controlled human infection models (CHIMs) involve intentionally infecting healthy volunteers with a pathogen as part of the trial design, typically to assess the efficacy of new vaccines and therapeutics [1]. The challenge agent may be a wild-type or attenuated pathogen. CHIMs have been conducted for many years and arguably have contributed valuable insights into disease pathogenesis and host responses to infection as well as accelerating development of novel therapeutics and vaccines [2].

There is an increasing recognition among key stakeholders that CHIM studies can play an important role in accelerating vaccine development. Nevertheless, the precise place within current product development and regulatory frameworks has yet to be clarified [3, 4]. The use of CHIM studies appears to have increased in the last 15 to 20 years [5], in particular in relation to the assessment of vaccines [2]. CHIM studies have been conducted in low-resource settings such as in Colombia [6, 7], Kenya [8], Tanzania [9], and Thailand [10]. Other settings such as India [11], Malawi [12], South America [13], and Africa more broadly [14] are being explored.

Despite their increasing use, there is very little specific guidance for CHIM studies. The World Health Organization (WHO) Guidelines on Clinical Evaluation of Vaccines: Regulatory Expectations 2016 [15]; the WHO Expert Committee on Biological Standardization 2016 [1] and 2017 [3]; and the Academy of Medical Sciences, Wellcome, and the Human Infection Challenge vaccine network 2018 [16] provide general guidance on CHIM studies. To the best of our knowledge, there are no specific guidelines relating to CHIMs in low-resource settings [12], although we acknowledge that the broad ethical principles remain consistent regardless of setting.

## Typhoidal *Salmonella* studies in Oxford

Enteric fever represents a major global health challenge. It is estimated to be responsible for about 12 million cases annually, predominantly in low- and middle-income countries [17–19]. The disease has been essentially eliminated as a public-health problem in high-income countries over the past century, largely owing to improvements in water quality, sanitation, and hygiene [20]. Long-term prevention of enteric fever will require improved access to safe drinking water combined with investment in sanitation and hygiene interventions. In the short to medium term, new control strategies for typhoid fever have arrived in the form of typhoid Vi-conjugate vaccines (TCVs), offering hope that disease control can be achieved in the near future. Despite this, several challenges remain, and in 2017 the WHO listed fluoroquinolone-resistant *Salmonella* as a “priority pathogen”, identified as one of 12 families of bacteria thought to pose the greatest risk to human health through rising antimicrobial resistance [21]. Since then, an extensively drug-resistant strain of S. Typhi H58 (combining a multidrug-resistant phenotype with exhibiting resistance to third-generation cephalosporins and fluroquinolones) has caused a large outbreak in Pakistan [22].

An extensive program of typhoid challenge studies was conducted in Maryland (USA) between 1952 and 1974, providing major insights into bacterial pathogenesis and acceleration of vaccine development [2, 23, 24]. This program eventually ceased, in part owing to a perception that conducting such studies in an institutional setting constituted an overly coercive environment despite the progressive ethical measures implemented by the investigators [2].

In recognition of the ongoing global challenge of typhoid fever, a program of typhoid and paratyphoid challenge studies was established at the University of Oxford from 2011 onwards. Since its inception, over 400 volunteers have been challenged in six separate studies in Oxford. Within one study, the leading typhoid conjugate vaccine in development (TypbarTCV®) has demonstrated an estimated efficacy of at least 55%, depending on the efficacy endpoint [25]. Data generated from the challenge model—coupled with data from previous typhoid conjugate vaccine trials, immunogenicity, epidemiological, and modelling data—have helped to advance the cause of typhoid conjugate vaccine deployment. This included a recommendation for programmatic use in a recent WHO position paper [26], WHO pre-qualification of the TypbarTCV® vaccine in early 2018 [27], and a funding commitment by Gavi, the Vaccine Alliance. Field trials of this vaccine in children are being conducted in Malawi [28], Nepal [29], and Bangladesh [30].

An advantage of CHIM studies is that they may be able to give data on the transferability of findings from the non-endemic to the endemic setting more quickly and cheaply than large field trials.

A detailed review of study design considerations, procedures, and clinical outcomes is beyond the scope of this report and these are reviewed elsewhere [24, 25, 31, 32]. However, ethical and practical issues arising from the studies in Oxford have not been specifically discussed in detail and therefore these are explored in this article. In addition, owing to the increasing interest in conducting CHIMs in endemic settings, we also address potential ethical issues of conducting similar studies in these settings.

## Ethical issues

We compare the following ethical issues between high- and low-resource settings: scientific justification, risk of harm to the individual and community, benefits to the individual and community, participant understanding, compensation, and regulatory requirements.

### Scientific justification in Oxford

Unlike non-typhoidal *Salmonella* serovars, *Salmonella* Typhi and Paratyphi are human-restricted pathogens. It remains unclear whether findings from challenge studies performed in non-endemic settings can be extrapolated to endemic settings, where the burden of invasive *Salmonell*a is highest. Findings from challenge studies in a highly selected group of volunteers in Oxford may differ from those found in endemic regions [8]. In the Oxford challenge studies, individuals who have lived in an endemic area for more than 6 months were excluded.

### Scientific justification in an endemic setting

Volunteers in endemic settings are likely to differ from UK volunteers across a range of important variables such as previous *Salmonella* exposure, diet, intestinal microbiota, and genetic profile [14]. For this reason, the use of CHIMs in endemic areas for vaccine development is being promoted. Volunteers in endemic regions may be genetically and immunologically more representative of the target audience for vaccine deployment. Consequently, efficacy results may be more representative of vaccine performance and the cost-effectiveness of deployment will be clearer to policy makers.

### Risk of harm to the individual in Oxford

The main concern in any research is risk of harm to participants [33]. In the *Salmonella* CHIM studies, healthy participants are deliberately exposed to *Salmonella* Typhi or Paratyphi. In the absence of vaccination, about two thirds of participants challenged will develop enteric fever. While the risks of enteric fever are low with prompt treatment, severe complications—including encephalopathy, intestinal perforation, and intestinal hemorrhage—can occur if left untreated or if infected with multidrug-resistant strains [34].

Several measures have been instituted to reduce the risk to participants, including the use of well-characterized and fully sensitive strains. In addition, stringent exclusion and inclusion criteria were applied to ensure that participants were healthy and would comply with study procedures [31]. Key exclusion criteria included significant medical, surgical, or psychiatric history.

The challenge studies performed in Oxford used an out-patient model, in which participants were resident in their own homes and attended the clinic site daily, albeit with strict hygiene precautions in place. After challenge, participants were reviewed that evening and then daily for 14 days. Physical observations and blood cultures were taken daily, and safety bloods and assessment of mental health were performed regularly. Symptoms were monitored daily with an online symptom diary. Participants had access to an on-call doctor 24/7 and provide a contact number for themselves and a second individual who could be called in the event that they could be contacted. They were aware that, if contact could not be made, they might be visited at home and that, in extreme circumstances, the police might need to be notified. They were instructed to inform the on-call doctor in the event of a persistent fever lasting 12 h. Participants meeting the criteria for enteric fever were seen in clinic and commenced on a 14-day course of antibiotics. Those not meeting the criteria received antibiotics regardless 14 days after challenge. Provision was made for direct medical admission in the event of severe enteric fever requiring in-patient management.

Participants were informed of prior experiences with human typhoid challenge, in which over 2000 volunteers have been exposed to live S. Typhi and have made a full recovery. We acknowledge that there is potential for anticipated and unanticipated serious adverse events to occur related to challenge. In order to capture such events, participants in early studies were followed up for a period of three years and for a minimum of 12 months in subsequent studies.

Participants were directed to their own general practitioner in the event of unexpected adverse events. The University of Oxford had appropriate insurance in place in the event of harm suffered as a direct consequence of participation in the study. Participants were advised to contact their private medical insurance provider (if they had one) before participating.

### Risk of harm to the individual in an endemic setting

As in non-endemic settings, in the absence of vaccination, about two thirds of participants challenged will develop enteric fever, which is higher than the daily risks in endemic settings. All measures taken in Oxford to reduce the risk to participants should also be instituted in endemic settings. In addition, safety measures should arguably be more stringent in settings where health systems and transport networks may be less robust. There must be appropriate hospital facilities available with prompt access to treatment, capacity for in-patient admission, and dedicated 24-h staffing. If transport networks are less reliable and pursuing incompliant participants would be difficult, an in-patient model might be safer.

To mitigate the risk of unexpected adverse events, there should be a minimum of enhanced surveillance and acces to enhanced care if challenge models were to be extended to low-resource settings.

Although the strains used in the Oxford studies were fully sensitive to fluoroquinolones, there remains a risk that additional use of these agents could drive by-stander resistance among other *Enterobacteriaceae* in healthy volunteers. The risk-benefit ratio would change in the context of endemic areas, specifically by balancing the need for treatment efficacy against driving further resistance. One approach might be to use alternative narrower-spectrum agents (e.g., trimethoprim-sulfamethoxazole or amoxicillin) in these settings.

### Risk of harm to the community in Oxford

A further ethical concern is the theoretical risk of harm to the wider community. Out-patient ambulatory challenge studies involving enteric pathogens pose a potential risk of transmission to study participants’ close contacts. The precise risk varies depending on the specific pathogen (e.g., *Salmonella* spp*.* vs. *Shigella* spp.) as well as local sanitation infrastructure, access to clean water, and effectiveness of hygiene interventions.

In the Oxford studies, it was assumed that sanitation infrastructure was adequate to prevent cross-contamination of sewage with drinking water. The risk of secondary transmission was mitigated by the application of strict hand-hygiene protocols. In part, this compelled us to include a few more exclusion criteria - participants who have high-risk occupations (as defined by Public Health England guidelines) [35], are immunocompromised or are in contact with vulnerable persons such as children under two years old, or have gallbladder disease, which can be associated with chronic typhoidal *Salmonella* carriage. Participants were also required to demonstrate clearance of *Salmonella* infection in three separate stool samples following antibiotic treatment. Household contacts were provided with written information about the study and offered screening if they wished. Participants were typically excluded if their contacts were unwilling to be exposed to the risk of transmission; this was the case for at least one individual within the Oxford studies. Early engagement during set-up was undertaken with Public Health England. In addition, local general practitioners and the wider public were informed of the studies.

### Risk of harm to the community in an endemic setting

In low-resource settings with less access to sewage treatment, there is a risk of transmission both to household contacts and to the wider community. Obtaining the consent of the wider community and contacts of study participants to accept this riks may be challenging and impractical. It might be necessary to conduct the study in an in-patient or residential setting but this would entail a prolonged stay of five or more weeks between challenge and confirmed clearance of the pathogen from stool.

If challenge studies were to take place in low-resource settings in an out-patient model, ideally there should be enhanced public health surveillance to trace any public health impact. Laboratory genotyping of typhoidal *Salmonella* strains from infected blood and stool from hospital in-patients during outbreaks would enable linking of outbreaks to challenge strains. Alternatives could include screening of household contacts or environmental surveillance or both.

### Benefit to the individual/community in Oxford

In the Oxford studies, the only benefit to the individual is vaccination with either a typhoidal vaccine or control vaccine. As volunteers in a non-endemic setting have a much lower risk of typhoidal *Salmonella* exposure (unless they travel to an endemic area), they were unlikely to benefit from any increase in immunity gained from typhoid vaccination or challenge. We did not systematically explore perceived benefits and motivations of participation, such as those related to altruism, experience, and finance [36, 37].

There was no community benefit beyond a broader contribution to the advancement of vaccine research. The vaccines assessed in this study could be used by travelers from high-income countries.

### Benefit to the individual/community within an endemic setting

Volunteers in endemic settings are more likely to benefit from any increase in immunity against typhoidal *Salmonella* that might arise through either vaccination or challenge. This provides additional justification to conduct research in the context in which it will be applied and in a community that stands to benefit the most from it. Consideration could be given toward providing enhanced levels of care, or health insurance, for the duration of follow-up.

### Participant understanding in Oxford

The informed consent process in CHIM studies must ensure that volunteers understand that their participation involves being deliberately infected with a disease-causing organism and that this may cause symptoms. Investigators must disclose all of the known potential risks of participating in a CHIM study so that potential volunteers can understand and make an informed decision regarding participation [38].

In the Oxford typhoidal *Salmonella* CHIM, participants must complete a pre-consent questionnaire to confirm understanding of the study, particularly the risks involved. If participants answered the questions incorrectly, the information was discussed again until the participant was able to retain and repeat the information correctly. Participants were excluded if they were unable to read the information booklet in English.

### Participant understanding in an endemic setting

Obtaining informed consent poses several additional challenges in low-resource settings, where participants are often less educated and less familiar with basic research concepts. In a malaria challenge study in Kenya, 100% of participants who came for screening took the quiz at least twice before “passing” the test [8].

To improve understanding, additional efforts (e.g., applying a multi-staged consent process and using multimedia to supplement written materials) must be made. This should be supported by extensive community and public engagement activities such as information sessions and community consultations. Researchers could adapt approaches used in other studies, such as mass drug administration studies, where community engagement is crucial [39].

### Compensation in Oxford

CHIM studies raise an important and long-standing ethical issue regarding the offering of appropriate compensation to participants. Careful consideration needs to be given to the level of financial compensation offered to participants in order to avoid undue inducement and blinding to the potential risks or harms of participation [40].

Volunteers in the Oxford CHIM studies are offered financial payments pro rata for time for visits, transport, blood sampling, and a presumed 10 days’ absence from work (whether volunteers were employed or not). Compensation for loss of work is 2.4 times that of hourly UK minimum wage for over-25-year-olds if an eight-hour day is assumed. In this study, the maximum amount of money paid to any participant is £3655. This amount is thought to be commensurate with the study demands, commitments, and burdens and was approved by the relevant ethics committees. We experience a fairly equal balance of student versus employed participants; a smaller proportion are self-employed, retired, or unemployed. The students have a greater flexibility of schedule to be available to undergo the challenge studies. The majority of them also have less recourse to paid work.

Potential participants could be excluded if the investigator felt that it was not in their interest to participate. Exclusion criteria were broad and included “a reason at the discretion of the study team”.

A number of persons of no fixed abode applied for screening but were mostly excluded on the basis of medical history of serious psychiatric disease and/or alcohol or illicit drug misuse. Some interested parties offered to fly from foreign countries and stay in hotels during the challenge period. An exclusion criterion was “residence in local area during challenge period”. Owing to the intensity of the study schedule, the need to be readily available for assessment, and length of follow-up, the researchers deemed that this was not in the participants’ best interest.

Where study investigators raised concerns regarding vulnerability or undue financial inducement, cases were discussed with senior investigators and, where appropriate, with their primary care clinician. In these instances, the decision to enroll volunteers was determined on a case-by-case basis.

### Compensation in endemic settings

Research in endemic settings often, but not always, involves inviting participants from low-resource settings. The amount of compensation given to participants in low-resource settings should be carefully considered. There are two concerns here: (1) undue influence, a situation in which an offer of something desirable influences decision-making in inappropriate ways [41], and (2) exploitation and injustice if the offer to prospective research participants is less because they are already impoverished.

Undue influence is considered unacceptable by most ethics committees. Mere influence (i.e., payment that may appropriately influence the decision to participate in scientifically valid research that has been approved by an ethics committee) is acceptable by most ethics committees [42]. The framework of Gelinas *et al*. distinguishes three rationales for payment: reimbursement for out-of-pocket expenses, compensation for time and burdens associated with research participation, and incentive to motivate participation. This approach offers a systematic way to calculate financial payments [42].

To ensure that compensation and incentives are appropriate for the context, consultation and engagement with relevant parties such as ethics communities and community advisory boards are essential [43]. It would seem reasonable to tailor compensations depending on local daily wage. Researchers should also learn from qualitative studies and the experience of others conducting CHIMs in low-resource settings [37].

### Regulatory requirements in Oxford

Regulatory requirements for challenge agents vary widely between countries [16]. Currently, stocks of typhoidal *Salmonella* challenge strains are manufactured in accordance with Good Manufacturing Practice standards to ensure safety and reproducibility. In addition, national and international ethical guidelines on human subject research apply; these include the Declaration of Helsinki [44], the Council for International Organizations of Medical Sciences guidelines [45], and International Conference of Harmonisation Good Clinical Practice [46].

### Regulatory requirements in endemic settings

If CHIMs were to be conducted in low-resource settings, the same quality standards should apply for the challenge strain. Considerations from production to administration are a particular logistical challenge. While imported stock will have a long supply line, a centrally manufactured product will maximize comparability between sites.

## Conclusions

Typhoidal *Salmonella* challenge studies in endemic settings are potentially highly valuable and feasible, providing a platform to address a major global health concern in the setting most affected. In addition to addressing country-specific host and environmental factors relevant to vaccine testing, the establishment of typhoidal *Salmonella* CHIM could increase research capacity, provide training of local staff in laboratory and research techniques, and provide a platform to undertake novel CHIM studies for other diseases. While typhoidal *Salmonella* CHIM studies are well established in the UK, trial protocols and regulatory processes must be adapted to local circumstances when transposed to an endemic setting. In-depth consultation and engagement with local stakeholders are required to address any potential concerns and to ensure ongoing ownership of the model. High levels of safety must be maintained to ensure confidence and to avoid reputational damage.

We conclude that, with careful consideration of country-specific ethical, practical, and regulatory issues, a typhoidal *Salmonella* CHIM in an endemic setting is both possible and desirable. It may be possible to phase out non-endemic CHIM studies once they can be performed safely and ethically in endemic settings.

## Data Availability

Not applicable.

## Abbreviations

CHIM: Controlled human infection model
TCV: Typhoid Vi-conjugate vaccine
WHO: World Health Organization
